# Ureteral Access Sheaths and Its Use in the Future: A Comprehensive Update Based on a Literature Review

**DOI:** 10.3390/jcm11175128

**Published:** 2022-08-31

**Authors:** Vincent De Coninck, Bhaskar Somani, Emre Tarik Sener, Esteban Emiliani, Mariela Corrales, Patrick Juliebø-Jones, Amelia Pietropaolo, Ioannis Mykoniatis, Belthangady M. Zeeshan Hameed, Francesco Esperto, Silvia Proietti, Olivier Traxer, Etienne Xavier Keller

**Affiliations:** 1 Department of Urology, AZ Klina, 2930 Brasschaat, Belgium; 2 Young Academic Urologists (YAU), Urolithiasis & Endourology Working Party, 6846 Arnhem, The Netherlands; 3 Progressive Endourological Association for Research and Leading Solutions (PEARLS), 75020 Paris, France; 4 Department of Urology, University Hospital Southampton, Southampton SO16 6YD, UK; 5 Department of Urology, Marmara University School of Medicine, Istanbul 34854, Turkey; 6 Department of Urology, Fundacio Puigvert, Autonomous University of Barcelona, 08025 Barcelona, Spain; 7 Service d’Urologie, AP-HP, Hôpital Tenon, Sorbonne Université, 75020 Paris, France; 8 GRC n°20, Groupe de Recherche Clinique sur la Lithiase Urinaire, Hôpital Tenon, Sorbonne Université, 75020 Paris, France; 9 Department of Urology, Haukeland University Hospital, N-5021 Bergen, Norway; 10 Department of Clinical Medicine, University of Bergen, N-5021 Bergen, Norway; 11 Department of Urology, Faculty of Health Sciences, School of Medicine, Aristotle University of Thessaloniki, 54124 Thessaloniki, Greece; 12 Department of Urology, Kasturba Medical College Manipal, Manipal Academy of Higher Education, Manipal 576104, India; 13 Department of Urology, Campus Bio-Medico University, 00128 Rome, Italy; 14 Unit of Urology, Magna Graecia University of Catanzaro, 88100 Catanzaro, Italy; 15 Department of Urology, IRCCS San Raffaele Hospital, 20132 Milan, Italy; 16 Department of Urology, University Hospital Zurich, University of Zurich, 8091 Zurich, Switzerland

**Keywords:** urolithiasis, nephrolithiasis, ureteroscopy, retrograde intrarenal surgery, ureteral access sheath, systematic review

## Abstract

Ureteral access sheaths (UASs) are part of urologist’s armamentarium when performing retrograde intrarenal surgery (RIRS). Recently, the world of RIRS has changed dramatically with the development of three game-changers: thulium fiber laser (TFL), smaller size single use digital flexible ureterosopes and intraoperative intrarenal pressure (IRP) measurement devices. We aimed to clarify the impact of UASs on IRP, complications and SFRs and put its indications in perspective of these three major technological improvements. A systematic review of the literature using the Medline, Scopus and Web of Science databases was performed by two authors and relevant studies were selected according to PRISMA guidelines. Recent studies showed that using a UAS lowers IRP and intrarenal temperature by increasing irrigation outflow during RIRS. Data on the impact of a UAS on SFRs, postoperative pain, risk of infectious complications, risk of ureteral strictures and risk of bladder recurrence of urothelial carcinoma after diagnostic RIRS were inconclusive. Prestenting for at least one week resulted in ureteral enlargement, while the influence of pre-operative administration of alpha-blockers was unclear. Since TFL, smaller single use digital ureteroscopes and devices with integrated pressure-measuring and aspiration technology seemed to increase SFRs and decrease pressure and temperature related complications, indications on the use of a UAS may decrease in the near future.

## 1. Introduction

Ureteral access sheaths (UASs) are part of urologist’s armamentarium when performing retrograde intrarenal surgery (RIRS). Until 2018, there was sufficient evidence that using a UAS increases irrigation outflow during RIRS and lowers intrarenal pressure (IRP) when using forced irrigation [1,2,3]. On the other hand, data on the reduced risk of infectious complications such as fever, urinary tract infection (UTI) and sepsis when using a UAS were conflicting. Data on the impact of a UAS on multiple reinsertions and withdrawals, stone-free rates (SFRs), ureteroscope protection or damage, postoperative pain, risk of ureteral strictures, as well as its cost-effectiveness were inconclusive [1,2].

Since 2018, the world of RIRS has changed dramatically with the development of three game-changers. Firstly, thulium fiber laser (TFL) became available as an innovative laser technology for the treatment of kidney stones [4]. Compared to Ho:YAG lasers, TFL seems to bring in many benefits including higher ablation speed, higher ablation efficiency, finer dust quality and reduced stone retropulsion [5]. Secondly, smaller size single use digital flexible ureterosopes became available on the market [6]. Finally, intrarenal pressure (IRP) measurement during RIRS was introduced in clinical practice, either with a wire including a pressure sensor [7] or with an integrated pressure-measuring suctioning UAS [8].

Considering the development of these three major technological improvements, it should be reassessed whether routine UAS placement is mandatory in contemporary RIRS or not. On this occasion, recent publications are reviewed to clarify the impact of UASs on IRP, temperature, SFRs, upper tract urinary carcinoma (UTUC) and complications including pain, infections, thermal injury and ureteral lesions [1]. Additionally, the influence of prestenting and alpha blockers on UAS placement needs to be clarified.

## 2. Methods

**Figure 1:**
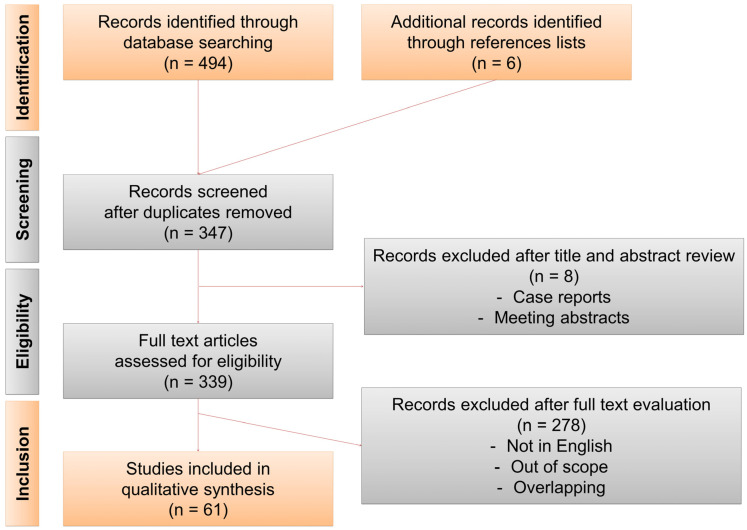
PRISMA flow diagram.

Two authors (V.D.C. and E.X.K.) conducted a systematic review of the literature using the Medline, Scopus and Web of Science databases in March 2022. The search terms (“ureter” OR “ureteral”) AND (“access sheath” OR “sheath”) AND (“ureteroscopy” OR “ureterorenoscopy” OR “retrograde intrarenal surgery”) were used and the filters “english” and “humans” were applied. Only articles published since October 2017 were considered, considering that prior articles had already been included in our previous systematic review [1]. Only studies relating to the role of UASs during RIRS for treatment of stones or UTUC were considered. Case reports, editorials and letters were excluded. Relevant studies were selected according to Preferred Reporting Items for Systematic Reviews and Meta-analyses (PRISMA) guidelines (Figure 1). Additional articles identified through references lists were also included. A narrative synthesis for analysis of the studies was used. To facilitate reading of and interpretation of the study results, all pressure values were converted to cmH_2_O. A factor of 1.35951 was used to convert mmHg to cmH_2_O.

## 3. Results

### 3.1. Intrarenal Pressure (IRP)

In the last few years, there has been an increased interest in evaluating IRP during RIRS. Doizi et al. evaluated IRP levels during flexible ureteroscopy, mini-PCNL, standard PCNL and endoscopic combined intrarenal surgery (ECIRS) in a kidney model [9]. Multiple irrigation pressures, multiple UASs and lithotripsy devices were evaluated. IRP never exceeded 50 cmH_2_O, even in the absence of a UAS, an empty ureteroscope’s working channel and an irrigation pressure of 193 cmH_2_O. A similar in vitro study focusing on flexible ureteroscopy was performed by Patel et al. [10]. In the absence of a UAS, they found that IRP surpassed 40 cmH_2_O at both 153 cmH_2_O and 193 cmH_2_O irrigation values, respectively. IRP exceeded 50 cmH_2_O in the absence of a UAS at 165 cmH_2_O. When using a UAS, IRP was below 40 cm H_2_O for all irrigation pressures.

Subsequently, Doizi et al. measured IRP during in vivo RIRS with laser lithotripsy in five cases with (n = 4) or without (n = 1) a UAS in place [7]. For the four cases with a UAS, mean baseline IRP, IRP with continuous irrigation at 80 cmH_2_O, during laser lithotripsy with on-demand forced irrigation and peak pressure were 5.8, 64.5, 115.1 and 367.2 cmH_2_O, respectively. For the case without a UAS nor prestenting, IRPs were 6.8, 57.1, 116.2 and 289.3 cm H_2_O, respectively [7]. Due to the small number of cases, conclusions cannot be drawn from comparisons between both groups. Similarly, Patel et al. prospectively evaluated IRP during stone lithotripsy in eight patients at the constant irrigation pressure of 204 cmH_2_O [11]. Pressure was measured in the renal pelvis, upper pole, interpolar and lower pole calyces both with and without a 12/14 Fr and 14/16 Fr UAS. Intracalyceal pressure was significantly lower in each region when a UAS was used. Compared to patients with a 12/14 Fr UAS, those with a 14/16 Fr UAS had significantly lower pressure in the interpolar (34.4 vs. 59.8 cmH_2_O) and lower pole (22.0 vs. 66.9 cmH_2_O) calyces. Interpolar calyceal pressure in the presence of a UAS was significantly higher than the IRP (41.9 vs. 24.3 cmH_2_O). Only with a 14/16 Fr UAS and their constant inflow pressure of 204 cmH_2_O, IRP and intracalyceal pressure did not exceed the threshold for renal backflow (40 cmH_2_O). Mean intrapelvic pressure in absence of a UAS was 56.1 cmH_2_O.

Loftus et al. investigated the effect of various IRP on histologic changes and fluid extravasation during simulated ureteroscopy in ex-vivo porcine kidneys. At pressure settings of 68, 136 and 272 cmH_2_O, mean percentage of renal tissue penetration was 33.1, 31.0 and 99.3% in the absence of a UAS and 0, 0 and 18.8% in the presence of a 12/14 Fr UAS, respectively [12]. Shresta et al. prospectively evaluated perirenal extravasation during RIRS in 71 patients. A 9.5/11.5 Fr or 10/12 Fr UAS could be placed in 60 (84.5%) patients. Irrigation was performed under gravity at 50 cm H_2_O with intermittent manual compression if required. Intraoperative perirenal extravasation of contrast was noted in eight (11.3%) patients. Extravasation was only associated with higher postoperative pain scores. It was not related to the use of a UAS, stone size, laser settings and duration, operative time and perioperative complications [13].

The effect of the ratio of the ureteroscope and the UAS diameter was evaluated by three groups [14,15,16]. They found that higher ratios were associated with higher IRP and subsequent increased resistive index in the arcuate artery. IRP was also strongly related to different irrigation devices. The ratio should be kept below 0.75 to maintain IRP below 40 cmH_2_O when using forced irrigation inflow pressures over 200 cmH_2_O.

### 3.2. Temperature

Fluid temperature increases proportional to laser power, irrespective of the ratio between energy and frequency. During laser activity, continuous irrigation flow should be applied to reduce the maximum temperature exposure. Importantly, it is crucial to maintain a balance between irrigation flow rates and power to avoid thermal damage besides pressure related complications [17].

Okhunov et al. evaluated temperature changes in TFL activation in a porcine kidney with and without a 12/14 Fr UAS in place [18]. Peak temperatures in the calices and at the tip of a flexible ureteroscope were measured during dusting (0.5 J, 80 Hz, 40 W) and fragmentation modes (1.0 J, 10 Hz, 10 W and 1.5 J, 20 Hz, 30 W), in the absence of stones. Both warm and room temperature irrigation fluid were used. Experiments were performed with a 7.5 Fr and dual lumen 9.9 Fr flexible ureteroscope. The authors found reduced or absent temperature spikes when using a UAS, room temperature irrigation, and/or a dual lumen ureteroscope [18]. Limitations to that study were the absence of a controlled irrigation fluid rate and the relatively high irrigation pressure (204 cmH_2_O), which is much higher than the commonly acknowledge cutoff for complications from high IRP. In another study, the association between heat production and Ho:YAG laser pulse modulations (length and type) was studied in a benchtop ureteral model using a 11/13 Fr UAS. Long pulse lengths produced significantly greater maximum temperature changes from baseline at 0.8 J/8 Hz and 1 J/10 Hz relative to Moses technology. The clinical relevance of these differences was questionable as the thermal dose for all pulse types remained under the threshold for injury throughout these low power settings. At higher power settings, the authors found no significant differences between pulse types [19]. Finally, Belle et al. compared risk of thermal injury between Ho:YAG and TFL generators and concluded that the TFL was associated with consistently higher heat generation. A limitation to this study is that the nominal laser power output was not controlled and may consequently have caused a systematical error to the study methodology [20].

### 3.3. Stone Free Rates

The main purpose of RIRS is rendering the patient stone free. Several groups recently investigated if SFRs are influenced by a UAS or not. Three groups found similar SFRs with or without a UAS [21,22,23]. Interestingly, two other groups found better SFRs in absence of a UAS. First, Meier et al. retrospectively investigated patterns of UAS use and associated outcomes across practices in 5316 ureteroscopy procedures. UASs were used in 1969 (37.7%) cases. After adjusting for clinical and surgical risk factors, UAS use decreased the odds of being stone free (OR = 0.75) [24]. Second, Kahraman et al. evaluated factors affecting the success of RIRS in 46 children. Better SFRs were achieved in the absence of a UAS, stones with HU < 700 and usage of a smaller flexible ureteroscope [25].

Komeya et al. investigated whether the gap between UAS and ureteroscope predicts SFR after RIRS with the fragmentation technique [26]. Gaps > 0.6 mm (>1.8 Fr), including the combination of a 9.5 Fr UAS and a small caliber ureteroscope, improved SFR and stone removal efficiency.

### 3.4. Complications

#### 3.4.1. Pain

Several recent studies evaluated postoperative pain complication when using a UAS. The Michigan Urological Surgery Improvement Collaborative (MUSIC) Reducing Operative Complications from Kidney Stones (ROCKS) initiative retrospectively investigated patterns of UAS use and associated outcomes across practices in 5316 ureteroscopy procedures. UASs were used in 1969 (37.7%) cases and were associated with increased odds of postoperative emergency department visits (OR = 1.50) and hospitalization (OR = 1.77) after adjusting for clinical and surgical risk factors [24]. Factors associated with post-ureteroscopy opioid prescriptions in 13,143 patients following RIRS included use of a UAS, besides year, younger age, male sex, higher BMI, absence of a pre-operative ureteral stent and stent placed during surgery [27]. These results are in contrast to the finding of a small prospective study evaluating postoperative pain complications when using a UAS (n = 30) or not (n = 30) during RIRS, in which no significant difference was found between both groups [21]. Similar finding were reported by Oguz et al. [28]. In another study, the size of the UAS was not correlated with postoperative pain [29].

#### 3.4.2. Infections

Several recent studies evaluated the risk of postoperative infections related to the use of a UAS. Bozzini et al. prospectively studied the impact of UAS use on postoperative infectious complications after RIRS [30]. UAS use was associated with less postoperative infections (16.3% vs. 37.1%). Use of UAS was associated with fewer cases of fever (15.2% vs. 32.6%, respectively), positive urine cultures (13.0% vs. 28.1%, respectively), positive blood cultures (3.2% vs. 13.5%, respectively) and urosepsis (2.2% vs. 8.9%, respectively) [30]. The authors did not comment on these very high rates of infectious complications in comparison to other research findings (0.2 to 15%) [31].

A retrospective study on risk factors for systemic inflammatory response syndrome (SIRS) by Mi et al. provide contrasting results. Out of 216 patients, 21 patients (9.7%) developed postoperative SIRS, with the use of an UAS as a predictor of postoperative SIRS (OR 6.1, *p* < 0.001), independently from urinary culture findings, stone size and surgery time [32]. Yitgin et al. evaluated outcomes of RIRS with (n = 51) or without (n = 62) a UAS, and did not find an association between UAS use and any kind of complications rate [22]. In a study evaluating preoperative and intraoperative risk factors associated with the incidence of SIRS, Inoue et al. found that a ureteral stent can be safely omitted when using 10/12 Fr UAS [29].

#### 3.4.3. Lesions

Bozzini et al. prospectively studied the influence of a 10/12 Fr UAS on intraoperative ureteral injuries during RIRS without prestenting. Even after stratifying data according to different post-ureteroscopic lesion scale (PULS) grades, they found no difference in ureteral injuries between the use of a UAS or not [30]. Similarly, Lildal et al. retrospectively evaluated the risk of ureteral lesions in RIRS with a 10/12 Fr UAS compared with an endoscope alone. Half of the patients in which a UAS was used were prestented. When adjusting for age and gender, the incidence of ureteral lesions was comparable between both groups [33]. These results contrast with the findings of Aykanat et al. who found less ureteral lesions with a smaller UAS (9.5/11.5 Fr vs. 12/14 Fr) in non-prestented patients in a well-conducted prospective study [34]. In case a 12/14 Fr UAS was used in patients without prestenting, Fully et al. recommended to measure the ureteral diameter preoperatively on a noncontrast CT with soft tissue window to predict the risk of ureteral injury by a UAS. This was based on their prospective study in 68 patients in which a smaller ureteral diameter was associated with an increased risk and severity of ureteral injury [35].

#### 3.4.4. Strictures

In 2019, long term follow-up results were published on the risk of strictures (ongoing hydronephrosis without an obstructing stone on follow-up imaging) following prospectively endoscopically identified high-grade ureteral injuries caused by a 12/14 Fr UAS. Only one out of the 56 patients developed a de novo ureteral stricture [36]. The authors concluded that endoscopically identified high-grade ureteral lesions following UAS placement do not lead to clinically significant sequelae on intermediate term follow-up, with a stricture rate comparable to those without visible injuries of 1.8%. Similarly, Aykanat et al. prospectively compared the risk of stricture formation one year after using a 9.5/11.5 Fr and 12/14 Fr UAS in non-prestented patients. Despite of an increased endoscopically identified risk of high-grade ureteral injuries with a 12/14 Fr UAS, there was no difference in ureteral stricture formation after one year follow-up [34]. Similar findings were published in two other retrospective studies on adults [37,38] and one in 48 children over a mean follow-up of 17 months [39].

This contrasts with results of a retrospective study on 1001 RIRSs in which stricture formation was diagnosed in 3% of patients after three months follow-up [40]. In multiple regression analysis, use of UAS was found to be an independent risk factor for stricture formation (OR = 4.6; *p* = 0.011), besides ureteral perforation (OR = 11.8; *p* < 0.0001) and surgical time > 60 min (OR = 5.7; *p* < 0.005) [40]. The authors did not comment on this high number of strictures compared to the literature [31].

### 3.5. UAS and the Three Game Changers

#### 3.5.1. TFL

Four clinical trials studying TFL lithotripsy were found [41,42,43,44]. All studies concluded that TFL is a safe and effective modality for lithotripsy during RIRS with minimal complication rates. In only one of these four studies, the use of a UAS was mentioned for all included patients [43]. Due to distinct variations in terms of stones location, size, density and laser settings between these studies, conclusions cannot be drawn about possible advantages or disadvantages of a UAS during TFL lithotripsy.

#### 3.5.2. Small Digital Flexible Ureteroscope

The smallest digital flexible ureteroscope on the market is the 7.5 Fr Uscope PU3033A (PUSEN™). This disposable instrument was evaluated in three publications [6,45,46]. As for most flexible ureteroscopes, it has a 3.6 Fr working channel [3]. Compared to the 9.5 Fr counterpart Uscope PU3022A (PUSEN™), it can be used within a smaller UAS without compromising on vision, deflection, maneuverability and safety [6]. Geavlete et al. evaluated the “no-touch technique” of this small ureteroscope for the treatment of renal stones in 24 patients. Access to the pelvicalyceal system was achieved in all patients without using a safety guidewire nor UAS. Ureteral inspection at the end of the procedure revealed no ureteral lesions [46]. In another study, Geavlete et al. compared the safety and efficacy of the “no-touch technique” (n = 144) in comparison of using a 12/14 Fr UAS (n = 144). Four flexible ureteroscopes were used, including the 7.5 Fr Uscope PU3033A (PUSEN™) in 14 patients. The “no-touch technique” resulted in higher success rates of access to the pelvicalyceal system (90.9% versus 83.3%) with less ureteral lesions (4.1% vs. 38.8%) [45]. Important limitations of this study were high risk bias associated with selection, performance detection, attrition and reporting due to its retrospective design, lack of randomization, incomplete outcome data on ureteroscope usage and no record of prestenting [47].

#### 3.5.3. Modified UAS with Pressure Control

In 2016, a modified 12/14 Fr UAS with an oblique suction-evacuation port with pressure regulating mechanism was presented by Zeng et al. In their proof-of-concept study, they found an improved stone clearance, improved visual field and reduced stone retropulsion owing to the new UAS [48]. This modified UAS concept, in which a UAS was connected to a suction machine, was also studied by Huang et al. in 2018. They observed high lithotripsy efficacy and low complication rates too [8].

In 2019, a thorough assessment of this concept was carried out in several studies. Chen et al. evaluated an integrated pressure-measuring suctioning UAS during RIRS for kidney stones between 2 and 3 cm [49]. Their intelligent pressure control was found to be safe and effective, with SFR comparable to minimally invasive percutaneous suctioning nephrolithotomy (93% vs. 95%; *p* > 0.05), and lower complication rates. In addition to this modified UAS, Deng et al. created an irrigation and suctioning platform as well as a UAS with a pressure-sensitive tip to record and monitor real-time IRP. Their pressure feedback technology was evaluated in 93 patients undergoing RIRS. They observed controlled IRP < 27 cmH_2_O with clear operative visualization [50]. This was confirmed by Chen et al. who investigated the safety and effectiveness of a novel flexible vacuum assisted UAS versus a traditional UAS in a porcine kidney model. They observed high stone volume clearance rates with IRPs < 10 cmH_2_O [51].

Two groups compared outcomes of RIRS with a traditional versus a suctioning UAS by connecting a channel on the tail of the suctioning UAS to a vacuum device. Suctioning UAS resulted in higher early SFRs [52,53], lower auxiliary procedure rates [52], lower incidence of infectious complications [53] and a shorter operative time [53].

### 3.6. Influence of Prestenting and Alpha Blockers

Two groups recently performed a meta-analysis to investigate the outcomes of pre-operative ureteral stenting for RIRS [54,55]. They both found that pre-stenting resulted in a higher success for UAS placement, lower intraoperative ureteric injuries and higher overall SFRs (defined as no residual fragment at all) [54,55]. In a recent study on porcine ureters, one week of prestenting resulted in a 3.8 Fr increase in the luminal diameter [56], as measured by xyz-methodology. Of interest, stent size (4.7 Fr vs. 7 Fr) and laterality (left vs. right) did not impact the degree of ureteral enlargement.

The impact of a three-day silodosin administration prior to surgery was evaluated in a prospective randomized trial on 77 patients undergoing RIRS with systematic use of a UAS and with no prestenting. Ureteral injury involving the smooth muscle layer (PULS Grade 3 to 5) occurred significantly less frequently in the silodosin group, compared to the control group (9.3% vs. 27.3%; *p* = 0.031). Patients who received silodosin before RIRS also reported significantly lower pain scores. There was no significant difference in the overall complications rate or SFR [57]. In a retrospective study on ureteral access in 76 non-prestented school-age children, tamsulosin did not facilitate successful ureteral orifice navigation after multivariate analysis [58]. In another retrospective study, 14 days of tamsulosin prior to RIRS did not improve the UAS placement rates [59].

### 3.7. UAS and UTUC

Douglawi et al. were the sole research group that recently examined the effect of diagnostic RIRS and UAS usage on bladder recurrence following radical nephroureterectomy for UTUC. They retrospectively reviewed 143 patients that underwent radical nephroureterectomy in which RIRS was performed beforehand in 104 cases (73%), of which a UAS was used in 26 (25%) patients. They found that a diagnostic RIRS in patients undergoing radical nephroureterectomy for UTUC significantly increased the risk of bladder recurrence (30.8% vs. 7.7%; *p* = 0.02). Multivariable analysis also revealed a significant increase in bladder recurrence if RIRS was performed prior to radical nephroureterectomy (HR 5.6). This effect was mitigated if a UAS was used (11.5% vs. 39.7%, *p* = 0.01) [60]. The authors did not mention whether an immediate instillation of intravesical chemotherapy following RIRS was administered or not.

## 4. Discussion

The recent literature confirmed findings that using a UAS lowers IRP and intrarenal temperature by increasing irrigation outflow during RIRS [7,9,10,11,12]. Data on the impact of a UAS on SFRs, postoperative pain, risk of infectious complications, risk of ureteral strictures and risk of bladder recurrence of urothelial carcinoma after diagnostic RIRS were inconclusive. Prestenting for at least one week resulted in ureteral enlargement. However, despite recent reports on the positive influence of pre-operative administration of alpha-blockers, evidence remains conflicting. All these discrepancies can be largely explained by the high risk of bias when scrutinizing all included studies [47].

Therefore, it should be questioned again whether we routinely need an UAS in daily practice for RIRS. In our opinion, placement of a UAS should not be a systematic step during RIRS. It may be reserved for difficult ureteral access or treatment of stone patients with an increased risk of infectious complications, as well as a possible addition in case of difficult operative conditions with low visibility due to low irrigation fluid outflow. Keeping the ratio of the ureteroscope and the UAS diameter below 0.75 seems an acceptable approach to maintain IRP below the threshold of pyelovenous backflow (40–60 cmH_2_O) when using forced irrigation inflow pressures over 200 cmH_2_O [14,15]. Although, this might be well influenced by the exact position of the proximal end of the UAS in the upper urinary tract [61].

Indications on the use of a UAS may decrease in the near future with the development of three game changers. Firstly, TFL allows finer stones dusting with higher ablation speed and ablation efficiency compared to Ho:YAG laser technology, without a significant impact on vision. This may result in less stone basketing with reduced number of reinsertions and withdrawals of a ureteroscope. Secondly, the downsizing of single use digital ureteroscopes undeniably facilitates access to the upper urinary tract. This may also reduce the rate of pre- and postoperative stenting. Downsizing ureteroscopies also results in more space between the outer surface of the flexible ureteroscope and the ureteral wall which improves outflow. This may reduce pressure and temperature related complications. Third, devices with integrated pressure-measuring and aspiration technology will undoubtedly shape the future of RIRS in terms of better SFRs, shorter operative time and again less pressure and temperature related complications. Therefore, future randomized prospective studies on TFL and the influence of downsizing ureteroscopes are warranted to confirm these hypotheses. Future developments should also focus on the development of ureteroscopes with an integrated pressure control system and active suction function to the UAS to leave no stone unturned and untreated.
